# The Self-Assembly of Lignin and Its Application in Nanoparticle Synthesis: A Short Review

**DOI:** 10.3390/nano9020243

**Published:** 2019-02-11

**Authors:** Pawan Kumar Mishra, Adam Ekielski

**Affiliations:** 1 Department of Wood Processing Technology, Mendel University in Brno, 61300 Brno, Czech Republic; 2 Department of Production Management and Engineering, Warsaw University Of Life Sciences, 02-787 Warsaw, Poland; adam_ekielski@sggw.pl

**Keywords:** lignin, self-assembly, noncovalent interactions, lignin nanoparticles

## Abstract

Lignin serves as a significant contributor to the natural stock of non-fossilized carbon, second only to cellulose in the biosphere. In this review article, we focus on the self-assembly properties of lignin and their contribution to its effective utilization and valorization. Traditionally, investigations on self-assembly properties of lignin have aimed at understanding the lignification process of the cell wall and using it for efficient delignification for commercial purposes. In recent years (mainly the last three years), an increased number of attempts and reports of technical-lignin nanostructure synthesis with controlled particle size and morphology have been published. This has renewed the interests in the self-assembly properties of technical lignins and their possible applications. Based on the sources and processing methods of lignin, there are significant differences between its structure and properties, which is the primary obstacle in the generalized understanding of the lignin structure and the lignification process occurring within cell walls. The reported studies are also specific to source and processing methods. This work has been divided into two parts. In the first part, the aggregation propensity of lignin based on type, source and extraction method, temperature, and pH of solution is discussed. This is followed by a critical overview of non-covalent interactions and their contribution to the self-associative properties of lignin. The role of self-assembly towards the understanding of xylogenesis and nanoparticle synthesis is also discussed. A particular emphasis is placed on the interaction and forces involved that are used to explain the self-association of lignin.

## 1. Introduction

Self-assembly is the process of formation of an organized structure or pattern from pre-existing disordered subunits, the condition being the non-involvement of external factors, driven only by internal forces and interaction occurring within the system. Based on the nature of subunits involved, it can be termed as molecular, supramolecular, or nanoparticle self-assembly. The main driving forces can be the attainment of equilibrium or minimization of free energy or inter-unit interactions; the inter-unit interactions are mostly non-covalent in nature.

Lignin, “nature’s glue,” along with hemicelluloses and cellulose micro-fibrils creates the mechanical backbone of vascular plants. The wood-derived lignin can be either of softwood or hardwood origin. Out of these two types, softwood lignin is composed primarily of coniferyl alcohol, condensed and found to be difficult to degrade. Additionally, it shows a relatively higher molecular mass and strong tendency to self-associate in a solution. The number of C-C bonds, 5’ linkages, β-β and β-5 bonds, cross-linking, and branching is also higher in softwood lignin, when compared to hardwood lignin [1]. These differences at structural, chemical, and compositional levels affect the noncovalent bonding and hence the source-dependent properties of the lignin [2].

**Figure 1:**
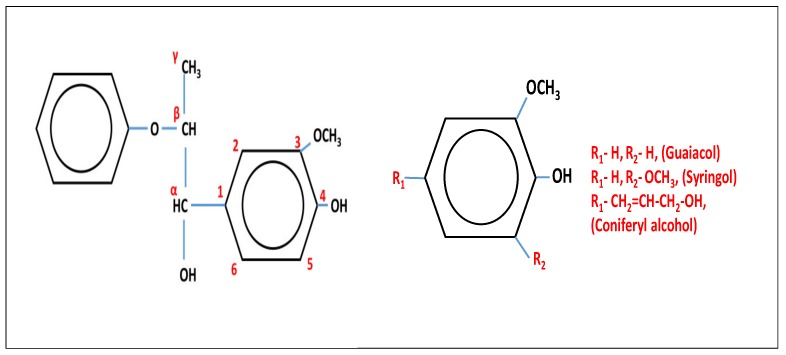
Basic structures and the standard numbering system used for molecules associated with lignin.

A basic structure to understand the atom numbering system in lignin is shown in Figure 1. In some cases, α, β, and γ are designated as 7, 8, and 9 positions, respectively. The random and chaotic linkage and branching in addition to supramolecular self-assembly provide lignin with its natural resistance against microbial and enzymatic degradation. On the other hand, this property of self-aggregation and the complex formation with other carbohydrate polymers (cellulose, hemicelluloses, and pectin), which leads to the formation of lignin carbohydrates complexes, create a major bottleneck in isolating pure lignin [3]. The lignin that is used in experiments or available as an industrial byproduct is different from native lignin, and their properties cannot be assumed as being entirely the same as the lignin in plant cell walls. These lignins are commonly termed as technical lignins. Their properties vary according to the source and processing method used for extraction.

**Figure 2:**
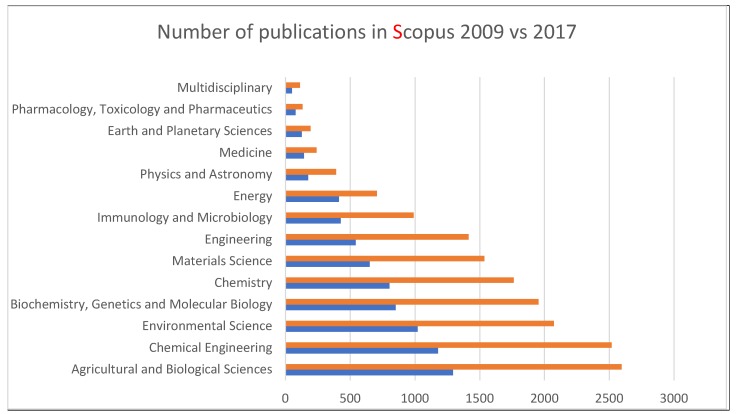
Distribution of the number of publications indexed in Scopus database, number returned using the keyword “lignin.”

The recent rise in reports of self-assembled lignin nanostructures with size tunability and morphology control adds a new dimension to the high-value applications of lignin [4,5,6,7]. The lignin nanostructure utilization in surfactants [8,9], UV protection [10,11], composites [12,13,14,15], and drug delivery [16,17,18,19] are among some of the latest reported studies (Figure 2). In this paper, we attempt to elucidate the progress in understanding 1) the process of lignin self-assembly in xylogenesis and in plant cell wall formation, 2) aggregation propensity and the effect of reaction conditions, 3) noncovalent interactions in molecular and supramolecular self-assembly, and 4) nanostructure formation.

## 2. Forces and Interactions

### 2.1. Aggregation Propensity and the Effect of Reaction Conditions

The tendency of lignin to self-assemble in a solution is very well documented. A few models and theories on this aspect have been proposed. The balance of electrostatic repulsion and van der Waals attraction (Derjaguin−Landau−Verwey−Overbeek theory) predicts the behavior of isolated lignin dissolved in a solvent. A number of parameters such as the nature and source of lignin, the pH of the solution [20,21,22,23,24], the temperature [25,26], and the nature of the solvent have also been studied. Investigations of the aggregation propensity or the self-assembly properties of lignin in a solution create essential data for understanding lignification, which in turn can be applied for delignification strategy development, nanoparticle synthesis, and biomimetic nanocomposite synthesis.

#### 2.1.1. Source and Extraction Processes of Lignin

The structure of lignin and/or derived technical lignin is central to the development of its valorization strategies [27]. Nanoparticle synthesis is one of many approaches to achieve this goal. Ideally, a well-defined native structure or with a minimized degree of condensation along with a minimal inorganic component should be the best form of lignin for nanoparticle synthesis. Clarity and homogeneity of the lignin structure will be highly desired to tweak and tune the properties of obtained nanoparticle from any kind of lignin. In this way, the requirements of lignin in nanoparticle synthesis are similar to those for efficient bio-refining approaches, i.e. lignin with minimized condensation and impurities [28]. However, despite the multitude of benefits of “lignin centric” approaches of pulping, it is still far from being an industrial reality [29]. In this paper, we exclude the strategies to retain the original structure and other bio-refining development for lignin valorization and keep our focus on nanoparticle synthesis from condensed technical lignin.

There are two aspects to the understanding of the difference in lignin behavior in solution based on the source and extraction process. First, the difference arising in the lignin structure and composition based on its source (soft wood, hardwood, and crop-based sources) [1]. Second, the lignin extraction from its natural source using some solvent involves bond breakage and hence dissolution. The process itself destroys the natural structure of lignin and leads to some degree of chemical modification; depending on the nature of solvent and process used, lignin undergoes a series of bond breakages and auto-condensations. Therefore, the family of “non-native” lignins with a respective influence of processing conditions is called technical lignin. Lignin in nature is formed by the polymerization of *p*-coumaryl alcohol, coniferyl alcohol, and sinapyl alcohol, bearing *p*-hydroxyphenyl (H), guaiacyl (G), and syringyl (S) units, respectively (Figure 1). Lignin based on herbaceous crops shows a higher number of H units. Gymnosperm-based (softwood) lignin lacks S units, while angiosperm-based (hardwood) lignin is rich in G and S. The structure of primary monomer affects the macromolecular structure (branching and cross-linking with the polysaccharides) as well as the degradability and processability of the source biomass [30,31,32,33].

The radical polymerization leads to several types of linkage formation, but the β-O-4 linkage is typically the most abundant. It is also one of the most easily cleaved bonds by most of the commonly used pretreatment methods. Hence, the β-O-4 content of extracted lignin is mostly affected by pretreatment and the severity of the methods. Sheng et al. observed that sulfonation lowered the surface energy of lignin and thus enhanced surface properties such as the van der waal interaction, Lewis acid base interactions, and the interaction of lignin with different ions [34]. In another study, Dawy et al. [35] (1997) reported the difference in intermolecular hydrogen bonding in samples of rice straw lignin, cotton straw lignin, and lignosulphonate lignin from bagasse. They also suggested a higher content methoxyl group in lignosulphonate as compared to other lignins (soda lignin from rice straw and cotton straw) and observed that many phenolic groups increased in lignosulphonate lignin by acid treatment as compared to the hydrogen peroxide treatment [35].

In a study by Constant et al. [28] (2016), six technical lignins covering three main industrial pulping methods (Indulin AT Kraft, Protobind 1000 soda lignin and Alcell, poplar, spruce, and wheat straw organosolv lignins) were comprehensively characterized by lignin composition analysis. The lignin molar mass was observed in the order: Indulin Kraft > soda P1000 > Alcell > OS-W ∼OS-P ∼OS-S, for all the methods used. Structural identification along with quantification of aromatic units and inter-unit linkages indicated that all types of lignin were degraded and condensed during processing [28].

In another study by Ratnaweera et al. [36] (2015), it was demonstrated that the assembly of lignin molecules in solvents varied based on the source of lignin and that the amount of methoxy groups present, i.e. the ability to crosslink, directly affected its associative propensities. The aggregates formed from softwood and hardwood lignins consisted of cylindrical blocks of 4–10 monolignol units, and the number of monomers per block depended on lignin concentration. These building blocks arranged themselves into large aggregates with well-defined shapes such as dense isotropic structures, random, network-like coils (interconnected nets), and two-dimensional nanomaterials depending on the source of lignin. It is notable that the lignin concentrations used in this work were above the overlap (threshold) concentration, which caused overlapping of pervaded volumes of the outermost chains, thereby resulting in the decrease in aggregate size with concentration for the loosely packed aggregates formed by both types (softwood and hardwood) of wood-based lignin. The densely packed lignin behaved in an opposite manner, wherein the lignin molecules associate to form larger aggregates with an increase in the concentration [36].

The hydroxyl group content directly affects the ability to crosslink, hence the associative propensity of lignin. The number of hydroxyl groups varies with the source and extraction process, as seen in Table 1. The source and extraction process of lignin directly affects the number of cross-linking sites (methoxyl and hydroxyl groups), hence affecting its aggregation intensity and tendency by affecting non-covalent interactions (the van der Waal interaction, Lewis acid base interactions, the interaction of lignin with different ions, and hydrogen bonding) that are seemingly the driving forces of the nucleation and growth of nanoparticles in a lignin solution.

#### 2.1.2. Nature and pH of Solvent

The solubility of polymer in a solvent involves chain disentanglement and solvent diffusion with a thermodynamically compatible solvent [38]. The solvent has mainly two roles in the context of this paper: first, bond cleavage and the dissolution of lignin from its natural source and, second, the nucleation and growth of the nanoparticle. Different solvents affect the nature and extent of bond breakage in different manners [39]. The availability of hydroxyl groups and lignin’s tendency to form hydrogen bonds makes it challenging to assemble its nanostructures in aqueous solutions [11,40]. However, utilizing the aqueous solution (can be acidic, alkaline, or neutral) as an anti-solvent (miscible with solution but does not dissolve the solute) is crucial for hydrophobic interactions that have been attributed as driving forces for lignin nanoparticle synthesis [41,42]. The dissolution of lignin in an aqueous solution of high pH can be attributed to the ionization of phenolic hydroxyl groups [43]. The effect of pH can be understood by its impact on this ionization, which in turn affects the electrostatic repulsion between similar charges—hence the associative behavior (hydrodynamic radius and self-assembly) of lignin.

The studies based on light scattering show self-association of Kraft lignin below pH 11.5, and pulse field gradient NMR data shows similar results below pH 9.0. Hysteresis explained the difference in the behavior of the protonation of the phenolic group, which depends on the condition that lignin is directly dissolved at a higher pH or that pH is increased gradually after dissolving the lignin at a lower pH. Additionally, on the basis of diffusion coefficients values, relatively little association was found to be occurring in a nonaqueous solvent in comparison to the aqueous solvent, which is attributed to the former’s low viscosity and increased translational mobility [44].

The additional role of pH is in the stabilization of nanoparticle suspension, which again can be attributed to the ionization of surface groups. In our experiment, we observed the role of alkaline pH-induced ionization and thereby the electrostatic repulsion responsible for the stability of the suspension [10]. In a study by Salenting and Schubert [45] (2017), the small angle X-ray scattering (SAXS) with model independent data analysis in combination with Dynamic Light Scattering (DLS) and Scanning Electron Microscopy (SEM), the colloidal transformations from ∼6 nm polymer assemblies of approximate ellipsoidal shape to well-defined submicron particles was observed with solvent change (THF—tetrahydrfuran) and pH. These authors reported it as a method to tune the synthesis of submicron particles using the variation in solvents and pH for high-value applications of lignin (drug delivery) [45]. In a similair study by Yang et al. [46] (2018), the solution struture of lignin in EG (ethylene glycol) and DMSO (dimethyl sulfoxide) were studied using small angle neutron scattering (SANS) and dynamic light scattering. They concluded that hydrogen bond formation is the main driving force lignin dissolution in EG, but it was not suggested to be necessary for dissolution in DMSO [46].

In a study by Sameni et al. [47] (2017), lignin–solvent interactions and lignin solubility in different organic solvents was studied using the solubility parameter (Hilderbrand theory) and the hydrogen bonding parameter (Hansen theory). According to the Hilderbrand theory, the closer the solubility parameter of solute and solvents, the better the solubility, and vice versa (compounds with good solubility in a solvent have a closer solubility paremeter). They observed that the solubility of lignin in different organic solvents cannot be acurately estimated using Hilderbrand solubility parameters. The maximum solubility of lignin was observed in pyridine and DMSO. The solubulity of lignin was also found to be dependent on molecular weight and the number of hydroxyl groups in lignin units [47].

A study of lignin chracteristics affecting its solubility aquaous solutions by Evstigneev [48] (2011) suggested that the solubility of lignin in aqueous alkali solutions is determined by the ratio of the number of phenolic hydroxyls (PHs) to the number of phenylpropane units (PPUs) in the lignin molecule. The minimal PH/PPU ratio for the dissolution of lignin was also reported to be 31:100 [48]. Based on the above-metioned reports and evidence, it can be comfortably hypothesized that the nature of a solvent affects the solvent–lignin interaction in the dissolution and agglomeration of lignin (hydrogen bonding in the case of EG and DMSO). Moreover, the role of pH in aqueous systems affecting the protonation and deprotonation (electrostatic interactions) of crosslinking sites and/or surface moities must be investigated and optimized for every method of lignin nanoparticle synthesis. Each case (type of techinical lignin) of precipitation (for naoparticle synthesis) needs to be studied and optimized in its specific and characteristic way.

#### 2.1.3. Effect of Temperature

The temperature as a major parameter for the supporting self-assembly of lignin has been less investigated. Still, some reports can be used for indirect evidence. A low temperature favors the self-association of lignin, and this was reported and used to explain low temperature-induced aggregation and lignin particle formation [10]. In another study, the unique property of lignosulphate to lose a surface charge in 0.1 M NaCl at 40 °C was explained by the presence of water molecules in two hydration shells—tightly bound inner shells and loosely bound outer shells—required for the group to retain charge; the increase in temperature caused the destruction of the outer hydration shell and hence a loss of charge [25]. This study also supported the role of temperature as a contributing factor to the lignin’s associative behavior in aqueous alkaline solutions. The solution behavior at different conditions and understanding of self-associative properties of lignin prepared the background for technical lignin-based nanostructure synthesis. The effect of temperature on self-assembly can be understood by the combined effect of the ionization constant (overall charge) and non-covalent interactions (hydrophobic interactions).

In a study on fractionation of technical lignins by Helander et al. [49] (2013), the separation of lignins from weak black liquor using a membrane with a cut-off of 1000 Da was performed. These authors concluded that the extraction of low molecular weight lignin could not be achieved at a higher temperature (70 °C, at pH 9) due to the formation of fairly lagre lignin agglomerates. However, lowering the pH to 7 and 4 led to finer particle formation, and some fractionation could be achieved. In addition to that, Generalized conclusions regarding the effect of temperature as a standalone factor on the self-associative properties of amphiphillic polymers were difficult to draw, as it always works in combination with other factors such as the pH of the soultion and the ionization contant of polymers [49].

### 2.2. Noncovalent Interactions and Self-Assembly

In the process of agglomerate formation and the stabilization of lignin, noncovalent interactions play a significant role. These interactions also affect the thermal properties of lignin, as strong interactions will reduce the thermal molecular motion of the lignin molecule. Associative properties of lignin are affected by the source of lignin and the nature of the solvent used, which can be understood by the difference in native structure and the composition of lignin. The solvent plays a central role in delignification of source-biomass by affecting bond cleavage and hence decides the chemical structure and the nature of the extracted lignin [39]. Although evidence of the self-assembly of lignin is available in both types of aqueous and non-aquaous media, the nature of forces working in different solvents cannot necessarily be assumed to be same [50]. Various forces are responsible for lignin aggregation in different solvents. Such forces can include, as evidenced by previous reports, π–π interactions, H bonding, van der Waals forces, and chain entanglement (in order of bond energy, van der Waals forces (<1 and 4 kJ·mol^−1^), hydrogen bonding (between 4 and 30 kJ·mol^−1^), and π–π interaction (between 4 and 30 kJ·mol^−1^) [51]).

#### π–π Stacking/Interactions

**Figure 3:**
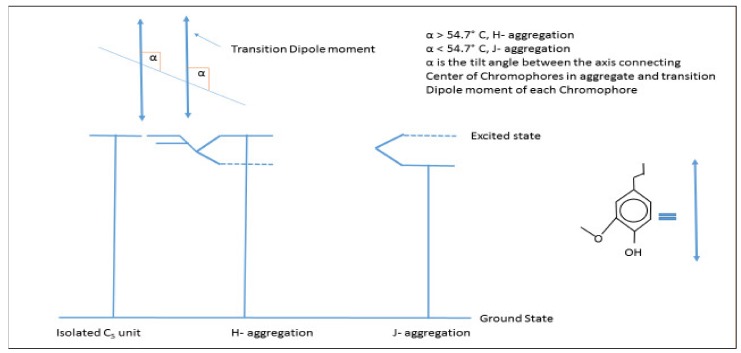
Representation of energy levels of H and J type aggregations [54].

These can be understood as forces of interaction between two aromatic rings, which are attractive in nature. This stacking is mainly favored in a face-centered configuration between electron-rich and electron-deficient aromatic groups, with exceptions to the large aromatic moieties [52]. In the case of lignin, the flat-disc shaped configuration of the lignin molecule in solution makes the case of self-assembly very specific; it allows simple π–π stacking and noncovalent bond formation. Based on the arrangement of aromatic groups, π–π stacking can be of two types: H type and J type (Figure 3). The H and J types of molecular assemblies are described by the tilt angle between the molecular axis connecting the chromophores and transition dipole moment of the chromophore. When this angle is less than 54.7 °C, the aromatic groups form J-aggregates with a bathochromic shift to the monomer absorption band. When this angle is more than 54.7 °C, the aromatic groups form an H–aggregate showing a hypsochromic shift to the M band [53]. In a study on sodium lignosulfonate, the formation of J-type aggregates and the π–π stacking of charge-free groups was confirmed [54].

In another study of the adsorption of lignin on the cationic surface, affinity coefficients were observed to be the same at all pH levels, supporting the dominance of π–cation interaction over the ionization hypothesis. In addition, π–π interactions also accounted for simultaneous self-assembly of solid or hollow particles of lignin [10,41,55]. As suggested by some reports, nanoparticles are formed by 10–15 nm subunits held together by non-covalent (π–π) interactions, and this assembly is reversible, thereby facilitating the potential role of lignin nanoparticles as a drug carrier for controlled drug delivery formulations [45].

#### 2.2.2. Hydrogen Bonding

Hydrogen bonding can be defined as the force of electrostatic attraction between two polar groups. A hydrogen atom that is covalently bonded to a highly electronegative atom nitrogen (N), oxygen (O), or fluorine (F) and is present in the field of other neighboring electronegative atoms forms these polar groups. Although no major study about the role of hydrogen bonding in molecular or supramolecular self-assembly of lignin was found in our literature survey, it is commonly cited as a contributing force due to its directional nature [24,56]. Additionally, the ability of the carboxylic group to form intermolecular hydrogen bonds has been reported to affect the mechanical properties of Kraft lignin-based gels [20]. In lignin agglomerates, both intermolecular and intramolecular H bonding can be expected to occur. An FTIR-based (Fourier transform infrared-based) study of lignin model compounds showed that aliphatic hydroxyl groups form stronger hydrogen bonds than phenolic hydroxyl groups. The dimeric biphenyl-type structure showed stronger intermolecular hydrogen bonds in comparison to other monomeric model compounds [57].

In a theoretical study on *p*-coumaryl β-O-4 model structure (2-phenoxy-1,3-propanediol), it was observed that intramolecular hydrogen bonding formed between α- and β-hydroxyl groups with the ether oxygen affects the bond order of aromatic carbon–oxygen bonds and thereby the rotational characteristics, hence affecting the preferred orientation of the molecule [58]. In another study, strong intermolecular hydrogen bonding was identified as the reason behind improved properties of lignin/poly(ethylene oxide) blends and the miscibility of both components [57,59].

In a recent study by Yang et al. [46] (2018), the solution structure of Kraft lignin was studied in EG and DMSO. They confirmed that hydrogen bonds are not necessary for the dissolution of Kraft lignin in DMSO. However, hydrogen bonding was found to be necessary for lignin dissolution in EG [46]. Hydrogen bonding has also been suggested as a mechanism of interaction between EG and lignin. The evidence in favor of this is based on the decrease in the solubility of lignin due to the acetylation of the hydroxyl and phenolic groups of lignin [60]. Among all methods reported for lignin nanoparticle synthesis, non-aqueous solvents such as THF, EG, or DMSO were used for preparing its solution. Among these studies, H bonding was never suggested to be responsible for Kraft lignin’s solubility in DMSO. Therefore, the contribution of H bonding in particle nucleation and growth in the lignin solution in DMSO can be assumed to be dissimilar to that in the lignin solutions of THF and EG.

#### 2.2.3. Chain Entanglement

The role of chain entanglement in the stabilization of supramolecular/three-dimensional network of the polymer can be understood as the physical cross-linking of non-covalently bonded polymer chains of smaller subunits [61]. Guerra et al. [50] provided the evidence in favor of the chain entanglement within the lignin macromolecule, wherein they observed a correlation in the total amount of β-aryl ether linkage and association/dissociation. They concluded that softwood lignins show a greater degree of association/dissociation as compared with hardwood and wheat straw lignin. They suggested that these association/dissociation phenomena are governed by chain entanglements operating within different macromolecules and intermolecular orbital interactions, dominated by those of the HOMO–LUMO type [50]. In the same study, an additional argument in support of intermolecular aggregation was provided by the adverse effect of iodine addition to acetobrominated lignin dissolved in THF. The same solvent, THF, has been used later in some studies for lignin nanoparticle synthesis using the solvent–anti-solvent route.

#### 2.2.4. Van der Waals Forces

Van der Waals forces are the weakest of the non-covalent interactions, resultant of attractive and repulsive forces. They can be further classified as permanent or momentary, on the basis of nature of dipole moments (London forces and diploe–dipole interactions). Van der Waals forces are commonly attributed as the reason for the self-assembly of different molecules [62,63]. Lindstrom and Westman reported a role of van der Waals forces in affecting lignin colloidal behavior especially in the case of macro-syneresis of Kraft lignin gel [64]. Strong van der Waals forces between lignin molecules and cellulose microfibrils were used as an explanation for strong adhesion between the lignin–carbohydrate complex and cellulose microfibrils [65]. Dipole–dipole interactions have been suggested to play a role in acetylated lignin–DMSO interactions. The carbonyl groups of acetylated lignin interact with DMSO via dipole–dipole interaction, not the hydrogen bonding (the absence of hydroxyl groups due to acetylation), as was evident in a previous study [47]. Due to the electrostatic nature of these forces, they can be assumed to be most affected by protonated and deprotonated states of lignin along with pH changes of the solution. Van der Waals forces are commonly thought to be responsible for the self-association of lignin in conditions that cannot be explained by hydrogen bonding.

#### 2.2.5. Hydrophobic Interactions

The hydrophobic interactions or forces represent the unusually strong attraction of hydrophobic groups in the presence of water, which cannot be explained using the “Lifshitz theory” of van der Waals forces. These forces can be qualitatively described as those responsible for the aggregation of hydrophobic moieties in the presence of water [66]. The hydrophobic interactions are commonly explained as forces of attraction within enzymes and proteins [67,68,69,70].

Different types of technical lignin show differences in hydrophobicity, as studied via hydrophobic interaction chromatography. The degree of hydrophobicity varies with the molecular weight of lignosulphonate and Kraft lignin fractions [71]. The presence of water remains the primary condition for these forces to be involved in self-assembly [41,42].

## 3. Nanoparticle Synthesis

Lignin self-assembly is key to understanding the chemical basis of xylogenesis in plant cell walls and nanostructure synthesis; hence, any discussion on the self-assembly of lignin without the inclusion of xylogenesis is incomplete. The challenge to understand a plant’s coordination methods of synthesis and the deposition of phenolic (hydrophobic) and carbohydrate (hydrophilic) biopolymers in the supramolecular organization of cell walls is still active [72,73]. From the developmental standpoint, lignification-initiating sites should be present in differentiating xylems before the start of the lignification process. These sites should be Ca-rich (needed for H_2_O_2_ formation with the help of enzymatically produced superoxide radicals), which is required for the polymerization of coniferyl alcohol [74].

The “outside in” pattern of lignin deposition in a maturing cell wall, where polysaccharides and proteins are already present, limits the structures that lignin can adopt. The self-assembly process of lignin deposition occurs in an aqueous environment and is directed by noncovalent interactions between monomers and dirigent proteins [75,76]. Another crucial aspect of xylogenesis and lignin assembly is substratum effects (lignin–carbohydrate and lignin–protein interactions) and their role in self-assembly.

Based on the fractal approach, some studies have already elucidated a supramolecular structure using the relationship between hydrodynamic and fractal properties of lignin [77,78]. The topological and supramolecular level fractal properties of lignin are due to non-linear self-assembly and are related to the dynamic mode of the strange attractors type of fractals [77,79]. As concluded in theoretical studies, if monolignols are assumed to diffuse to the site of lignification, then the fractal theory can be utilized in explaining the biosynthesis, polymerization, and molecular and supramolecular assembly of lignin [78]. Dynamic self-organization has been reported in the enzymatic dehydrogenation polymerization of monolignols in in vitro lignin biosynthesis, and the Belousov–Zhabotinskii system has been used to study them. Additionally, macromolecular parameters of softwood lignin correspond to the universal Witten–Sander class of fractals. On the other hand, the softwood lignin structure is related to a star-like structure whose scaling parameters are determined by the linear topology of branches [77].

The forces and mechanisms involved in lignin nanoparticle formation follow the same trend as the self-associative properties mentioned above, wherein the solvent, the nature and the source of lignin, and the temperature used in the study play a crucial role. The forces involved in the formation and stabilization of unmodified lignin-based nanostructures fulfill their part at two stages: nucleation and growth (condensation and coagulation). In the case of precipitation using an anti-solvent, the rate of nucleation of a nanoparticle depends on the degree of super-saturation during the mixing process of a solvent and an anti-solvent. This degree of super-saturation varies locally until the mixing process is complete (Matteucci et al. [80] 2006). The rate of primary nucleation depends on some parameters and are explained by a number of equations. Followed by nucleation, particle growth occurs by condensation (diffusion and incorporation of the molecule to the particle surface by non-covalent interactions) and coagulation [80]. The coagulation negatively affects the rate of condensation by decreasing the total number of particles and hence the surface area. The rate of nucleation has a direct contribution to the formation of fine particles, especially if the rate of particle growth can be controlled. In Table 2, key information from some of the latest reports of nanoparticle formation is listed. A similar mechanism can be understood by the solvent–anti-solvent method reported for lignin nanoparticle formation [55,81].

These results are analogous to our observations where we suggested the one drop-one particle hypothesis for the nanoparticle formation based on D–lignin (dioxane soluble fraction of Kraft lignin) [10]. In our unreported study, we managed to synthesize monodisperse nanoparticles of alkali lignin using aerosol-assisted self-assembly. This is in contrast to our published study, in which dioxane soluble fraction of alkali lignin was used and resultant particles showed a significant level of polydispersity [10]. The role of homogenization as an additional force to assist hydrophilic–lipophilic interactions in the nucleation of organosolv lignin residue-based nanoparticles was clearly demonstrated by Rao et al. [86].

In a report by Xiong et al., the control of morphology was made possible for enzymatic hydrolytic lignin by varying the temperature and the rate of solvent–anti-solvent interaction (15 °C and 4 mL/min of the anti-solvent rate for solid particles but 25 °C and 2 mL/min for the single hole particles) [41,42]. They also suggested the role of π–π interactions and hydrophobic interactions for particle formation. Their study involved de-ionized water as an anti-solvent, which is a prerequisite for hydrophobic interactions to come into play. In a recent review, lignin nanostructure synthesis, hydrogen-bonding, hydrophobic interactions, layer-by-layer self-assembly, and solvent-induced π–π interactions were suggested as possible mechanisms behind the molecular and supramolecular self-assembly of lignin [45].

For cases of hollow particle formation, a list of methods is given (Table 2). A nanoemulsion-based soft template mechanism has been reported by Xiong et al. [83]. They suggested the phase separation and formation of a membrane layer at the interface of THF and water, leading to the wrapping of water, which is later evaporated and results in the formation of hollow particles or capsules. In this study, the authors suggested a role of hydrophobic interactions in the formation and stabilization of hollow nano-spheres. In another study, ultrasonication was used to promote the hydrophobic interactions and shell formation. Ultrasound-assisted cavitation, radical generation, and emulsification were identified as underlying phenomena [83]. In our study, we suggested the peripheral precipitation based on the difference in the time of diffusion and the time of the freezing of the solvent as a responsible factor for hollow particle formation. Additionally, as the whole process occurred in a non-aqueous environment, the contribution of hydrophobic interaction was ruled out. The difference in the dynamics of lignin in a droplet concerning the time of freezing and the time of diffusion played a significant role [10].

## 4. Conclusion

The integration process of hydrophobic lignin in plant cell walls, while the nature of the reaction mixture changes from hydrophilic to hydrophobic, is still not fully understood, and some theories have been proposed to explain it. However, a clear role of non-covalent interactions and self-assembly can be accepted in the process of lignification. The contribution of fractal theory in assigning different fractal natures to softwood and hardwood lignin has also helped in understanding lignification and self-assembly at various scales. In the case of technical lignin and its self-assembly in ex-situ conditions, the derived knowledge is hard to generalize due to the complex chemical structure of lignin. A major bottleneck is created by source and extraction process dependent variation in the structure of lignin. This is in addition to the variations observed due to the nature of the solvent, pH, and the temperature of assembly conditions. Nevertheless, studies on the role of hydrogen bonding, hydrophobic interactions, and π–π interactions in aqueous conditions, and that of inter/intramolecular hydrogen bonding and solvent modified π–π interactions in organic solvents, can be found in the literature. A partial role of chain entanglement has also been suggested. As attempts to synthesize tailor-made nanostructures of lignin increase over time, understanding of the ex-situ self-assembly of technical lignin and possibly the process of lignification will improve with time. An understanding of self-assembly properties and contributing factors is of utmost importance for lignin valorization. Most of the high value applications of lignin and its resource utilization are based on the knowledge of lignificantion/xylogenesis and nanostructure syntheis. The effective, commercially viable, and environmentally benign processes of delignification cannot be developed without a clear understandng of xylogenesis, where self assembly properties in addition to lignin interaction with other carbohydrates and proteins play a central role. Similiarly, lignin nanostructure synthesis for possible applications in UV protection, biomedical applications and biomimetic nanocomposite synthesis utilizes the self-aggregation propensity of lignin as a pricncipal driving force. The effective utilization of lignin offers multiple benefits such as biomass valorization and waste utilization. First results seem very promising; however, due to a limted number of studies on biocompatibility, toxicity, drug/gene delivery, and other biomedical applications, the full potential of this underutilized biomaterial still needs to be explored. In the field of lignin-based biomimetic composites, some studies are available, but more research needs to be done before the final product hits the market.

## Figures and Tables

**Table 1:** Total hydroxyl group content of lignin (mmol·g^−1^) based on different sources and methods of extraction.

Type	Source	Phenolic	Aliphatic	Carboxylic	Total
Soda lignin	Wheat straw	2.29	2.1	1.10	5.49 ^a^
P1000 soda lignin	Sarkanda grass and wheat straw	1.85	1.79	1.11	4.75 ^a^
Alcell/organosolv ^#^	Mixed hardwoods (maple, birch and poplar)	1.91	1.11	0.29	3.31 ^a^
Kraft/indulin AT ^#^	Softwood	2.31	2.41	0.54	5.26 ^a^
Alkali pretreated	Wheat straw	1.58	1.07	0.92	3.57 ^a^
Biolignin/organic acid extracted	Wheat straw	1.0	3.9	-	4.9 ^b^
Organosolv ^#^	Hardwood	4.0	1.5	0.1	5.6 ^b^
Kraft ^#^	Softwood	3.4	1.7	0.0	5.1 ^b^

^a^ [37], ^b^ [36], ^#^ different suppliers.

**Table 2:** Information from some of the latest reports on the synthesis of hollow nanoparticles/nanocapsules of lignin.

Type of Lignin Used and Structure Formed	Method Used	Mechanism Suggested	Reported by
Ultrasonic Assisted Synthesis			
Dioxane soluble fragment of alkali lignin, hollow colloid	Ultrasonic spray-freezing	Peripheral precipitation	[10]
Kraft lignin, microcapsules	Ultrasound driven self- association	Ultrasound-mediated cross-linking	[82]
Kraft lignin, nanocapsules	Ultrasonication of microemulsion	Ultrasound-induced self-assembly and complexation	[83]
Kraft lignin, hollow nanospheres	Ultrasonication assisted solubilization, anti-solvent addition	Self-assembly at two different phases	[84]
Wheatgrass lignin and Sarkanda grass lignin	Ultrasonication of aqueous suspension	Side chain cleavage/depolymerization and oxidative coupling/polymerization	[85]
**Homogenization assisted synthesis**			
Organosolv lignin residue, submicron spheres	Homogenization enhanced nucleation	Hydrophilic-lipophilic aggregation	[86]
**Change of pH and/or solvent**			
Enzymatic hydrolytic lignin, single hole nanospheres	Solvent-anti-solvent precipitation method	Layer by layer self- assembly outside to inside	[42]
Kraft lignin, nanocapsules	Solvent-anti-solvent precipitation method	Spontaneous distribution of hydrophilic-lipophilic sequences	[87]
Softwood Kraft lignin, lignin Nanoparticles	Anti-solvent addition using dialysis bag	Chemical precipitation	[55]
lignin nanocontainer	Interfacial polyaddition	Polyaddition reaction at interface	[88]
Enzymatic hydrolytic lignin, solid nanospheres	Solvent–anti-solvent precipitation method	Layer by layer self-assembly inside to outside	[41]
Kraft lignin, nanospheres	Solvent-anti-solvent precipitation method	Chemical precipitation	[89]
Low sulfonated lignin (indulin AT)	Solvent-anti-solvent and base-acid precipitation	Chemical precipitation	[81]
Kraft lignin	DMF (solvent)-compressed CO_2_ (anti-solvent)	Expansion by anti-solvent	[90]
